# Tetraspanins in the regulation of mast cell function

**DOI:** 10.1007/s00430-020-00679-x

**Published:** 2020-06-07

**Authors:** Zane Orinska, Philipp M. Hagemann, Ivana Halova, Petr Draber

**Affiliations:** 1 Division of Experimental Pneumology, Research Center Borstel, Leibniz Lungenzentrum, Airway Research Center North, German Center for Lung Research (DZL), Borstel, Germany; 2 Department of Signal Transduction, Institute of Molecular Genetics of the Czech Academy of Sciences, Prague, Czech Republic

**Keywords:** Tetraspanins, Mast cells, FcεRI, Mast cell degranulation, Allergy, Antiviral immune response, Exosomes

## Abstract

Mast cells (MCs) are long-living immune cells highly specialized in the storage and release of different biologically active compounds and are involved in the regulation of innate and adaptive immunity. MC degranulation and replacement of MC granules are accompanied by active membrane remodelling. Tetraspanins represent an evolutionary conserved family of transmembrane proteins. By interacting with lipids and other membrane and intracellular proteins, they are involved in organisation of membrane protein complexes and act as “molecular facilitators” connecting extracellular and cytoplasmic signaling elements. MCs express different tetraspanins and MC degranulation is accompanied by changes in membrane organisation. Therefore, tetraspanins are very likely involved in the regulation of MC exocytosis and membrane reorganisation after degranulation. Antiviral response and production of exosomes are further aspects of MC function characterized by dynamic changes of membrane organization. In this review, we pay a particular attention to tetraspanin gene expression in different human and murine MC populations, discuss tetraspanin involvement in regulation of key MC signaling complexes, and analyze the potential contribution of tetraspanins to MC antiviral response and exosome production. In-depth knowledge of tetraspanin-mediated molecular mechanisms involved in different aspects of the regulation of MC response will be beneficial for patients with allergies, characterized by overwhelming MC reactions.

## Introduction

Mast cells (MCs) are long-living cells highly specialized in the storage and release of different biologically active compounds. Located in different tissues and organs in the proximity of blood vessels and nerves, MCs quickly respond to changes in the environment by granule exocytosis and release of mediators. Organ-restricted local reactions relevant for the specific function (e.g., local extravasation, bronchoconstriction) or generalized systemic reactions (e.g., changes in blood pressure during anaphylactic shock) are the consequences of MC response. Mainly associated with different pathological conditions such as allergies or asthma, MCs are able to recognize pathogens and regulate local and systemic inflammation in the context of protective immune reaction [1–4]. As cells sensing the environment on one hand and changes in organism homeostasis on the other one, MCs display heterogeneity in granule composition and organization of membrane complexes [5, 6]. Different developmental origin [7, 8] and maturation under the influence of tissue-specific microenvironment are at least two factors responsible for MC heterogeneity and tissue-specific MC features. MC degranulation starts by receptor-mediated incoming signals followed by reorganisation of granules and actin cytoskeleton [9]. These processes end up in granule movement to the cell surface, granule membrane fusion with plasma membrane, and granule content release. IgE-dependent MC degranulation is an interplay between the high affinity IgE Fc receptor (FcεRI) complex, activator and inhibitory proteins, membrane lipids, downstream tyrosine kinases, cytoskeletal proteins, as well as proteins and lipids involved in granule membrane organization [10–12]. Behind IgE-antigen binding, MC degranulation could be induced by proteases (e.g., thrombin), danger signals (e.g., ATP), toxins, small polycationic compounds, and neuropeptides [13, 14]. Tetraspanins—an evolutionary conserved family of transmembrane proteins—are involved in organisation of membrane protein complexes. Therefore, tetraspanins probably play an important role in the regulation of MC function, particularly, in regulation of exocytosis and membrane reorganisation during the degranulation and after degranulation is completed.

## Introduction to the tetraspanin family of proteins

Tetraspanins are membrane glycoproteins consisting of 204–393 amino acids with four conserved transmembrane helices, a small extracellular loop EC1 (9–26 amino acids long), and a large extracellular loop EC2 (up to 138 amino acids long) [15–17]. The EC2, being responsible for binding partner proteins, contains a conserved Cys–Cys–Gly amino acid motif (CCG-motif), two other conserved cysteines, and up to four additional cysteines [18]. The intracellular N- and C-termini are usually short. Characteristic post-translational modifications of tetraspanins are *N*-glycosylation (at asparagines) at the EC2, palmitoylation (at cysteines), and ubiquitination (at lysines) [18]. Cholesterol binding is a general feature of tetraspanins, since 30 out of 33 human tetraspanins contain at least one cholesterol-binding motif [19]. Interaction between tetraspanins and cholesterol is necessary and sufficient in the formation of migrasomes—migration-dependent membrane-bound cellular organelles [20]. Cholesterol binding was verified after solving the structure of CD81 [21]. The crystal structure of CD81 also indicates that EC2 exists in an open and a closed conformation [21]. Tetraspanins were thought to be mixed together in tetraspanin-enriched microdomains, but super-resolution data indicate TEMs consisting only of one type of tetraspanin [22]. Whether tetraspanin distribution in membranes, particularly in immune cells, is cell-type specific is unknown. It is also unclear whether tetraspanin distribution differs between normal and malignant cells. Such effects have been described, e.g., for organisation of B-cell receptor complex [23].

In humans, 33 tetraspanins have been identified so far. The nomenclature of genes and proteins of the tetraspanin family could be found at the HUGO Gene Nomenclature web page (www.genenames.org). According to phylogenetic analysis, the tetraspanin family members can be subdivided into four groups: the CD family, the CD63 family, the uroplakin family, and the Retinal Degradation Slow (RDS) family [24]. Huang et al. suggested that tetraspanin families including CD9/CD81/TSPAN2 and CD37/CD82 are produced by en bloc duplications [25]. Therefore, it is possible that tetraspanins can compensate the absence of each other, leading to mild phenotypes of single tetraspanin knockout mice.

Palmitoylation is the post-translational modification where palmitoyl-CoA is enzymatically bound to a thiol group of a cysteine [26]. For tetraspanins, this seems to take place mainly in the Golgi complex, but modifications at the plasma membrane are also very likely [27]. A recent study from Rodenburg et al. shows that palmitoylation is a stochastic process rather than being defined by distinct motifs [28]. All tetraspanin proteins can be palmitoylated. Tetraspanins with mutated palmitoylation sites show decreased association with their interaction partners including cholesterol [18, 29].

Ubiquitination is a process by which ubiquitin is enzymatically coupled to free lysine residues on proteins. Besides signaling, poly-ubiquitination leads to protein degradation in the proteasome. The Single Subunit Transmembrane E3 Ligase Gene Related to Anergy in Lymphocytes (GRAIL) can ubiquitinate tetraspanins, specifically at the N-terminus by binding the EC2 of the tetraspanin [30]. Ubiquitination of Tspan6 is critical for interaction with mitochondrial antiviral signaling (MAVS) and recruitment of downstream signaling proteins [31].

Glycosylation of tetraspanins at asparagines in the EC2 is common. Loss of glycosylation leads to multiple effects, e.g., loss of glycosylation in CD151 affects glycosylation of the binding partner of CD151, integrin α3β1 [32]. Likewise, in B cells, CD81 is required for CD19 surface expression and proper glycosylation of CD19 depends on CD81 in the Golgi complex [33].

Recently, it was found that the tetraspanin CD37 themselves can take part in signal transduction and could be phosphorylated at the N- and C-termini [34]. Whether this observation is human CD37-specific or B-cell chronic leukemia cell-restricted, does phosphorylation take place in other tetraspanins bearing identical or similar ITIM-like motifs and whether phosphorylation affects interaction with other membrane proteins, should be further investigated.

Altogether, tetraspanins as an evolutionary old membrane protein family are involved in a variety of different cellular processes such as cell development, motility, fusion, and cancer development [18]. Direct association of target proteins with several tetraspanin partners suggests that the function of the target protein could be dependent on associated tetraspanins [35]. Interacting with other membrane proteins, e.g., integrins, G protein-coupled receptors (GPCRs), a disintegrin and metalloproteases (ADAMs), tetraspanins are organizing membranes in functional microdomains [36] and as organizers “they are making the impossible processes possible and the possible—more efficient and adaptable” [25].

## Tetraspanins in MCs

Tetraspanins were among the first human MC receptors that have been extensively studied by the group of Peter Valent during the late 1990s in human MCs isolated from different organs and tissues. Using different monoclonal antibodies, the tetraspanins CD9, CD81, CD82, CD63, and CD151 were detected on the surface of human mast cells [37–40]. Experimental findings describing effects of tetraspanin-specific antibodies on MC activation, degranulation, and migration, present for CD9, CD81, and CD63, and functional changes in murine MCs, deficient for tetraspanins CD63 and CD151, have been extensively reviewed elsewhere [41–44]. Therefore, in this review, we paid a particular attention to tetraspanin gene expression in different human and murine MC populations, discussed tetraspanin involvement in regulation of key MC signaling complexes, and analyzed potential contribution of tetraspanins to MC antiviral response and exosome production.

**Fig. 1:**
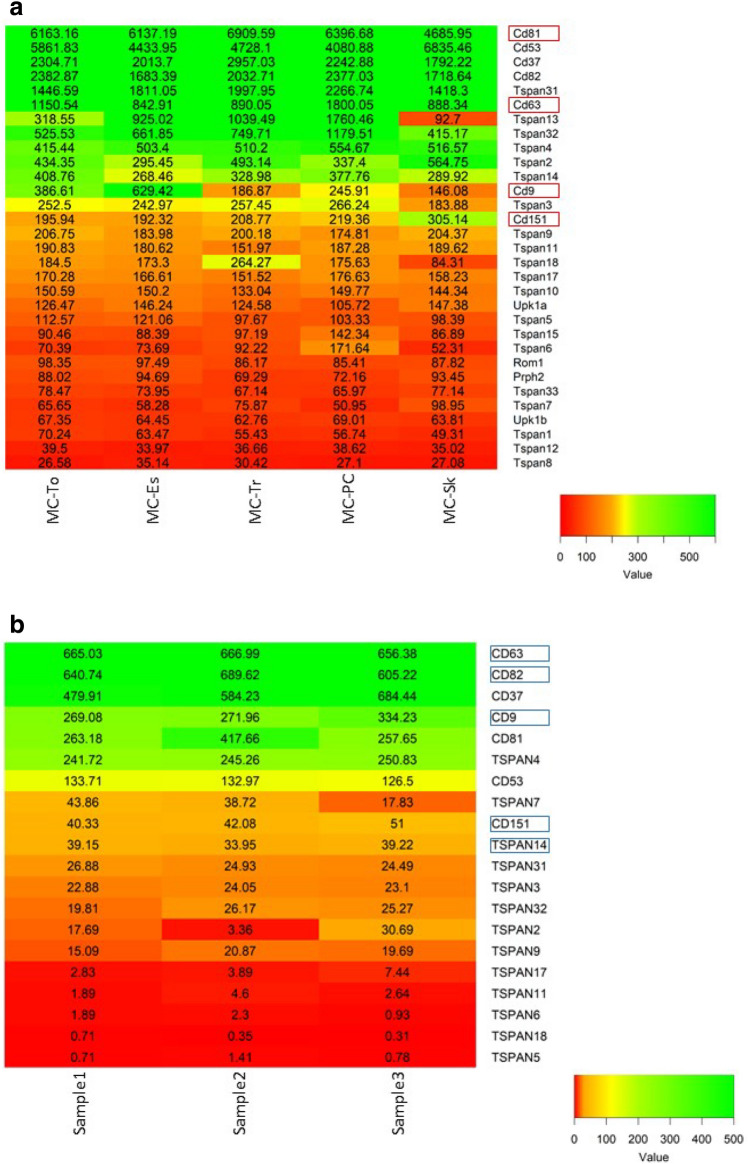
Expression of tetraspanins in murine and human MCs. **a** Tetraspanin expression in different murine MC subsets. Gene expression was analyzed in following MC populations-tongue MCs (MC-To), esophagus MCs (MC-Es), trachea MCs (MC-Tr), peritoneal MCs (MC-PC), and skin MCs (MC-Sk) [45]. The heat map was generated with data from http://www.immgen.org/ choosing MCs as the cells of interest and using r gplot heatmap2. The gene expression level was determined by Affymetrix microarrays (GEO: GSE377448). Tetraspanins involved in the regulation of MC function are outlined. **b** Expression of tetraspanin genes in ex vivo human skin MCs isolated from three different donors. Expression data were generated by cap analysis of gene expression (CAGE) technology within the FANTOM5 project and published as Supplemental Table 3 in [61]. Plotting the extracted tetraspanin gene expression data was performed with r gplot heatmap2.  Tetraspanins identified by skin MC proteome analysis [62] are outlined

Gene expression data recently generated by the ImmGen consortium provide gene expression signature of mouse MCs isolated from skin, peritoneal cavity, trachea, tongue, and esophagus. At the same time, these data enabled us to obtain an overview of gene expression of almost all tetraspanin family members [45]. As shown in Fig. 1, CD81, CD53, CD37, CD82, and Tspan31 are the MC tetraspanins with highest expression, followed by CD63, Tspan13, Tspan32, Tspan4, Tspan2, Tspan14, CD9, Tspan3, and CD151 showing intermediate expression levels. Tspan9, Tspan11, Tspan18, Tspan17, Tspan10, Upk1a, and Tspan5 are expressed at low levels. Tetraspanin Tspan13, Tspan32, and CD9 expression varies between different MC populations, indicating that tissue-specific factors could affect tetraspanin expression patterns. Peritoneal MCs and skin MCs showed the highest degree of differential gene expression, differing also in the observed tetraspanin pattern. CD53, Tspan2 and CD151 expression is higher in skin MCs as compared to other MC populations. Interestingly, also in bone marrow-derived mast cells (BMMCs) tetraspanins Tspan32, CD53, and CD82 belong to stimuli-specific transcriptional signature and are upregulated upon IL-33/IgE, IL-33, and IgE stimulation, respectively [46]. It is unknown how the tetraspanin pattern changes upon MC activation and how the changes of one tetraspanin expression influence the expression level of other tetraspanins and their interaction partners. This has been described, e.g., for TspanC8 tetraspanins and ADAM10 [47].

While the function of CD63, CD81, CD9, and CD151 in MCs has been intensively examined either by antibody effects or by genetic models [48–52], the role of CD37, CD53, and CD82 has been only poorly investigated so far. Interestingly, many tetraspanins, expressed in different immune cells and involved in the regulation of different signaling pathways, have not been analyzed at all, regarding their potential effects on MCs.

Tspan31, Tspan13, Tspan32, Tspan4, Tspan2, Tspan14, and Tspan3 have not been described in MCs so far. TSPAN31 is a tetraspanin highly expressed in human rhabdomyosarcoma cell line RH-30. As a natural antisense transcript of cyclin-dependent kinase 4 (CDK4), TSPAN31 regulates the expression of CDK4 mRNA and protein in hepatocellular carcinoma cells (HCC) [53]. By controlling Akt signaling pathway, TSPAN31 is involved in the control of cell survival and cell motility [53]. TSPAN13 is tetraspanin mainly localized in cytoplasm and nucleus and associated with different types of malignancies [54]. Plasmacytoid dendritic cells and naïve B cells are human immune cells expressing TSPAN13 [55]. In mouse, in addition, thymocytes and small intestine lamina propria macrophages are expressing high Tspan13 mRNA levels [56]. Tspan32 is highly expressed in naïve CD4^+^ T cells, NK cells, ILC3s, and different B-cell subpopulations [56]. In human cells TSPAN32 is mainly located in the nucleoplasm [57]. Tspan4 is highly expressed in monocytes and dendritic cells [56] and is essential for migrasome formation [20]. TSPAN2 is responsible for invasion and motility of different cancer cells and TSPAN2 interaction with CD44 is crucial for maintenance of the intracellular level of reactive oxygen species [58]. Tspan14—a tetraspanin of the TspanC8 subgroup—regulates ADAM10 activity [59] and is highly expressed in murine eosinophils, neutrophils, hematopoetic stem cells, pre B cells, and B1a cells [56]. Tspan3 responsible for migration and proliferation of oligodendrocytes [60] is expressed in dendritic cell populations [56]. Further investigations will prove whether MC tetraspanin gene expression data will correspond to the presence of proteins and to which extent these tetraspanins are involved in the regulation of MC function.

Comprehensive analysis of human skin mast cell transcriptome was performed by the FANTOM consortium analyzing human skin MC gene expression in freshly isolated, in vitro expanded, and in vitro expanded/stimulated cells [61]. From 33 tetraspanins described, expression of 20 tetraspanins was detected in human skin MCs (Fig. 1). CD63 and CD82 were the tetraspanins with the highest expression, followed by CD37, CD9, CD81, TSPAN4, and CD53. Proteome analysis of skin MCs performed by Gschwandtner et al. detected CD63, CD82, TSPAN14, CD9, and CD151 [62]. However, none of the tetraspanins was exclusively expressed in MCs. Single-cell analysis combined with quantitative proteomics and analysis of post-translational modifications of tetraspanins will improve the understanding of tetraspanin involvement in regulation of MC function.

## Tetraspanin effects on key MC signal receptor complexes

MCs are main effector cells in allergies. Extensive degranulation and release of preformed mediators—protective in response against helminths or hemathophageous ectoparasites—are detrimental in the pathogenesis of allergic diseases [63]. Binding of allergen-specific IgE to FcεRI is a pre-requisite for allergen-induced degranulation. Pseudo-allergic IgE-independent reactions against polycationic compounds—such as non-steroidal neuromuscular blocking drugs or antibiotics of fluoroquinolone family—are the second type of hyper-responsivity leading to MC degranulation [64]. This type of MC degranulation is mediated by Mas-related G protein-coupled receptor b2 (Mrgprb2) in mice and its human orthologue MRGPRX2 [64].

There are experimental evidences, indicating that tetraspanins are involved in the modulation of FcεRI-mediated MC activation. Initial studies, highlighting tetraspanins as important regulators of MC function, started with the observation that CD81- or CD63-specific monoclonal antibodies are inhibiting IgE-dependent mast cell degranulation in vivo [48, 49]. In vitro effects studied in rat basophilic leukemia cells indicate that early FcεRI-mediated signals such as tyrosine phosphorylation and Ca^2+^ influx were not affected by antibody treatment. Instead, treatment with CD63 specific antibodies influenced PI3K pathway, involved in regulation of adhesion and degranulation. CD9-specific antibodies were enhancing Ca^2+^ influx, tyrosine phosphorylation of non-T-cell activation linker (NTAL), and MC degranulation. CD9 colocalization with FcεRI was shown by electron microscopy of isolated plasma membrane sheets [50]. The association of CD81 and CD9 with trimeric FcεRI was described in human monocytes [65] and association of CD9 with Fcγ in macrophages [66]. Whether CD9, CD63, CD81, and CD151 interact with FcεRI complex proteins directly or whether they are recruited upon FcεRI-mediated activation remains unclear. One of the early events in FcεRI-mediated signal transduction is the change of intracellular Ca^2+^ concentrations. Therefore, Tspan18, modulating activity of Ca^2+^ channel Orai1 in endothelial cells [67], could be involved in the modulation of MC degranulation, as well. The mechanism of how exactly antibodies against tetraspanins modulate MCs and basophil activation and migration have not yet been sufficiently clarified. An important role here could play a crosstalk between tetraspanins and integrins as well-known tetraspanin interaction partners [68]. MCs and basophils express a variety of integrins on their surface [69]. However, the exact role of integrins in MCs activation remains enigmatic. Adhesion of BMMCs to fibrinogen-coated plates enhanced cytokine production and degranulation upon IgE/Ag stimulation or SCF-induced activation [70, 71], but the role of tetraspanins in this process was not studied. Further studies of tetraspanins in the regulation of FcεRI-mediated signals will help to understand the function of individual tetraspanins and tetraspanin microdomains.

Whether tetraspanins are involved in the regulation of Mrgprb2-mediated MC degranulation is another open, unresolved question. The involvement of CD81 in the regulation of GPR56 function in NK cells [72] is another example of how tetraspanin is contributing to GPCR mediated signaling. Also finding that tetraspanin CD151 is upregulated in asthma patients led to discovery that CD151 regulates airway smooth muscle cell contractions mediated by GPCR activation through regulation of intracellular calcium release and its involvement in protein kinase C translocation to the membrane [73].

**Fig. 2:**
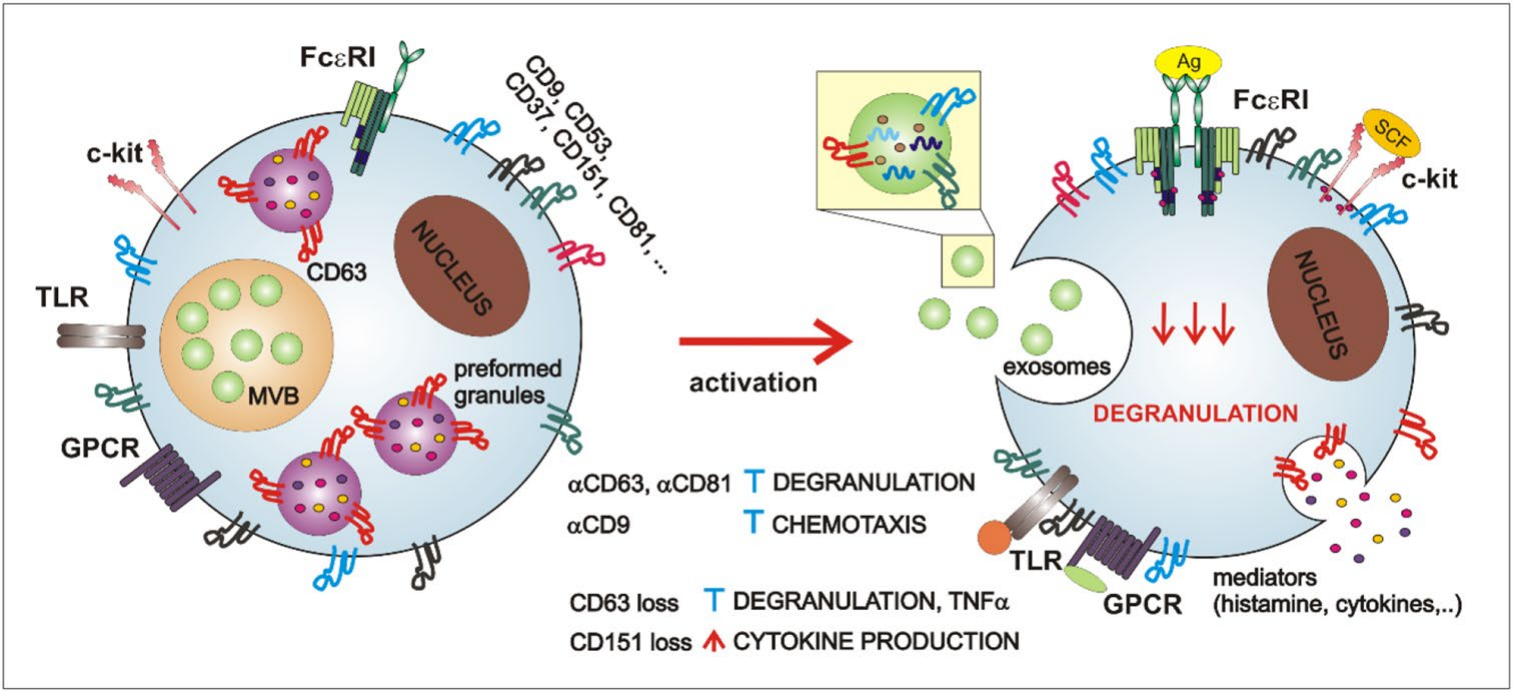
Different tetraspanins in non-activated and activated MCs. Majority of tetraspanins is expressed on MCs surface with exception of CD63 that is expressed also on MCs preformed granules in non-activated MCs. MCs contain preformed granules and form multi-vesicular bodies that give rise to exosomes. Stimulation with IgE/Ag complexes leads to release of several mediators including histamine, proteases, cytokines, as well as exosomes and, probably, redistribution of tetraspanins. Whether MC tetraspanins are involved in regulation of c-kit, TLR, or GPCR-mediated signals is unknown. MCs’ exosomes contain several proteins, RNAs and DNAs and tetraspanins CD9, CD63, and CD81 were identified on their surface. Antibodies against several tetraspanins were found to inhibit degranulation (αCD63 and αCD81) or chemotaxis (αCD9). On the other hand, loss of CD63 leads to decreased degranulation and TNFα release, whereas loss of CD151 potentiates cytokine production

Membrane receptor tyrosine kinase c-kit signaling is essential for both human and mouse MC development, survival, maturation, and activation [74]. c-kit ligand stem cell factor (SCF) strongly enhances MC degranulation acting synergistically with FcεRI [75]. However, changes in cytoskeletal organization caused by chronic SCF exposure markedly impaired FcεRI-mediated degranulation [76]. c-kit interaction with tetraspanins is described in myeloid cell line MO7e and hematopoietic stem cells. Stimulation with c-kit ligand SCF is required for c-kit colocalization with CD9, CD63, and CD81 [77]. Studies of c-kit effects in tetraspanin-sufficient and tetraspanin-deficient MCs will help to understand whether tetraspanin -c-kit interactions are cell-type specific and functionally relevant. Since c-kit could be shedded from the membrane, interaction of TspanC8 tetraspanin subgroup with ADAM10 is another potential mechanism, how tetraspanins can modulate c-kit signaling and function [78]. MC-specific ADAM10 deficiency leads to suppression of SCF-induced MC migration, but enhances proliferation and survival [79]. Interestingly, vice versa c-kit is affecting the expression of tetraspanins, since overexpression of mutated D816V c-kit protein in Baf3 cells induces histamine production and expression of CD63 and CD53 [80]. Tetraspanin effects in regulation of key MC signal receptor complexes are summarized in Fig. 2.

Since MC activation could lead to MC degranulation, an identification of reliable MC activation markers is important diagnostic issue. Biomarkers of activated MCs and basophils can be divided into two main subgroups: released allergy mediators (e.g., histamine, heparin, peptidases, and chymases) and surface markers of cell activation, represented by proteins with different membrane expression in activated vs. non-activated cells [81, 82]. Tetraspanin CD63 and ecto-nucleotide pyrophosphatase/phosphodiesterase CD203c are the best characterized membrane biomarkers and both are routinely used in human allergy diagnostic as a part of the basophil activation test (BAT). However, both have their limitations and the search for new activation-relevant MC and basophil markers is ongoing. Tetraspanin CD63 acts as “all-or-nothing” biomarker in basophils. Non-activated basophils express no surface CD63, whereas activated cells have maximum surface expression of CD63 which could be easily detected by specific antibodies using flow cytometry [83, 84]. The situation is different in MCs where even non-activated MCs express a significant amount of CD63 on their surfaces; however, like in basophils, its amount increases upon IgE-Ag activation [85, 86]. Cell surface expression of CD63 is negatively regulated by TM4SF1 protein and positively regulated by cytoplasmic protein syntenin-1 [87]. Later one is highly expressed in MCs [56]. Here, it should be mentioned that at least two different isoforms of CD63 were identified in human mast cells and specific monoclonal antibodies were able to identify granular form of CD63 exposing C170 and N172 as critical determinants for antibody binding only upon IgE-Ag stimulation [88]. Antibodies recognizing granular CD63 isoform were able to block MC degranulation upon repeated IgE/Ag stimulation [88]. CD203c is the biomarker commonly used together with tetraspanin CD63. It is expressed in small amounts also on the surface of non-activated MCs and basophils and similarly to CD63 significantly upregulated upon MCs and basophil stimulation [89]. However, the dynamics of CD203c and CD63 membrane up-regulation are different and probably induced by different signaling pathways [90, 91]. Also the stability of CD63 and CD203c on plasma membrane depends on cell isolation and storage procedures [90]. Both activation markers should be used complementary in combination with transmembrane proteins with stable expression in activated and non-activated cells such as CCR3, CD123, and others (reviewed in [81]). Although expression of tetraspanins, such as Tspan32, CD53, and CD82 could be changed upon activation [46], whether these proteins could be used as MC activation markers should be further investigated. Much less is known how CD63 as MC granule membrane protein affects granule content. In neutrophils, CD63 was found to be involved in targeting of neutrophil elastase precursor (proNE) into intracellular granules. A direct association between CD63 and proNE has been shown upon coexpression in COS cells [92]. Cell-type-specific or protease-specific effect cannot be formally excluded, since granule morphology and cathepsin D content were not affected in CD63 deficient BMMCs [51].

## Tetraspanins in regulation of MC antiviral response

The immune response to viral infections is a complex and dynamic process. It involves an antiviral response on targeted surfaces acting in concert with various components of the host immune system. Innate and adaptive effectors react at different stages of infection to different pathophysiological changes induced by the invading viruses [93, 94]. Since MCs are tissue-resident sensory cells, their participation in antiviral response becomes more and more evident. MC response can have an impact on antiviral resistance, tolerance or immunopathology, and tissue damage [95]. Beyond their direct antiviral effects, MCs could participate in antiviral reactions by the recruitment and conditioning of other effector cells. Tetraspanins are membrane proteins frequently used by viruses for entering, traversing, and exiting cells during the viral infections [96, 97]. CD81 is characterized as receptor for hepatitis C and binds E2 viral glycoprotein [98]. CD9, CD81, CD82, and Tspan7 might be incorporated in the HIV-1 virus [99] and CD63 is important for HIV-1 reverse transcription [100]. MCs express different receptors involved in the recognition of viral RNAs—Toll-like receptors (TLRs), retinoic acid-inducible gene 1 (RIG-1), and melanoma differentiation-associated gene 5 (MDA5) [101, 102]. Khan et al. recently characterized CD82 as the TLR9 interacting partner in macrophages [103]. Association of CD82 and TLR9 takes place in ER. TLR9 trafficking to CpG-containing endolysosomal compartments and TLR9-mediated signaling, NF-κB activation, and TNFα production are controlled by CD82. Furthermore, RIG-1-mediated signaling is regulated by Tspan6, which interacts with MAVS and negatively regulates downstream signaling pathway [31]. However, it is unclear whether, also in MCs, CD82 and Tspan6 are involved in TLR9 and RIG-1 regulation. Association of CD81 with SAM And HD Domain Containing Deoxynucleoside Triphosphate Triphosphohydrolase 1 (SAMHD1) controls subcellular localisation of the hydrolase and the metabolic rate of HIV replication by controlling dNTP availability [104]. MCs are characterized as inducible reservoir for HIV-1 infection [105] and are able to capture HIV-1 and to pass on to T cells [106]. CD81 is highly expressed in MCs and SAMHD1 has been detected in MCs and MC-derived exosomes [56, 107]. Therefore, CD81 participation in modulation of MC antiviral response should be an object of further investigations. Concluding, tetraspanins could be involved in the control of MC antiviral response and could modulate both viral replication and host immune response.

## Tetraspanins, exosomes, and mast cells

Exosomes are nanovesicles of endocytic origin with diameters of 30–100 nm. They are formed either by reverse budding of peripheral membrane of multi-vesicular bodies or late exosomes [108, 109]. Biological properties and physiological functions of exosomes were extensively reviewed recently [109, 110], and many important and updated information can be found in public online databases such as Vesiclepedia (www.microvesicles.org) [111] and ExoCarta (www.exocarta.org) [112]. Vukman et al. comprehensively outlined MC-specific exosome features [113]. In the following, we will summarize key findings regarding MC exosomes and tetraspanins.

The majority of cell types produce exosomes; their content and role are distinct for each cell type [114]. In mast cells, exosomes are stored in intracellular granules and could be released upon mast cell activation [115, 116]. The ability of BMMCs and MC lines P815 and MC/9 to secrete exosomes was proved in 2001 [116]. These immunologically active exosomes were able to induce blast formation, proliferation, and cytokine production of B and T cells [117]. In contrast to mast cell lines that produced exosomes spontaneously, pretreatment with IL-4 was necessary for exosome secretion by BMMCs. Similar potential to induce splenocyte activation upon IL-4 stimulation was also detected in mature peritoneal mast cell [118]. The interest in exosomes increased when it was shown that these nanovesicles can serve as vehicles for the transfer of information (proteins, lipids, and RNA) between individual cell types [107, 119]. The group of Jan Lötvall demonstrated that exosomes produced by murine mast cells did not contain only proteins and lipids but also both mRNA and microRNA. Secreted exosomes were taken up by human mast cell line and murine proteins were then expressed in recipient cells [107]. The composition of exosomes may vary depending on the state and mode of MC activation [115, 116, 120, 121]. Groot Kormelink et al. showed that depending on the MC activation status, distinct subsets of extracellular vesicles are released [122]. They differ in phospholipid composition, protease activity, and tetraspanin content, and could possess differential immunological functions. MC exosomes can contain MHC class II molecules, FcεRI complex proteins, c-kit tyrosine kinase, MRGX2, chemokine receptor CCR1, and tryptase [115, 123–126]. Tetraspanins, namely CD9, CD63, and CD81, are widely expressed in extracellular vesicle subpopulations and are often used as an exosome markers (Fig. 2). However, their presence was also identified in apoptotic bodies and microvesicles which are other types of extracellular vesicles [127]. Although, according to Vesiclepedia, tetraspanins are among the 50 top extracellular vesicles-expressed proteins (microvesicles.org) and are highly expressed in mast cells [42], their role in mast cell exosome formation and function is still unknown. Surprisingly, information about the presence of different tetraspanins in mast cell exosomes is also very limited.

A tetraspanin that is mainly connected with mast cell exosomes is CD63 which is often used as a marker during exosome purification [116, 122, 128, 129]. Tetraspanin CD63 is mainly expressed at the membrane of secretory lysosomes, including serotonin-containing granules that, during activation, fuse with the plasma membrane [51, 85], and therefore, it is not surprising that CD63 is found also on exosomes. The presence of the tetraspanin CD63 and, in some cases, also CD37 was identified in exosomes produced by mast cell line MC/9. Other tetraspanins, such as CD9 and CD81, which have been previously identified in exosomes produced by other cell types, were not identified by this screen. On the other hand, isolation and characterization of CD63 positive exosomes produced by human mast cell line HMC-1 revealed the presence of tetraspanins CD9 and CD81 [127–129]. Involvement of tetraspanins in exosome protein assembly, recruitment of microRNA and micronuclear DNA in exosomes, selection of exosome target cells, and the uptake of exosomes by the target cells are only some aspects underlining the significant potential of tetraspanins as targets for therapeutic interventions [130–132].

We would like to emphasize that research on the mechanisms of exosome production and uptake is still in its infancy. However, the mere fact that MC exosomes are able to transmit information to both mast cells and other cell types suggests that there is an enormous potential in exosome research. Given the significant presence of tetraspanins on exosome surface, it is indisputable that tetraspanins will play a key role in future research.

## Conclusions and outlook

Remarkably that more than 20 years after the description of tetraspanins in MC membranes, our knowledge about the processes regulated by tetraspanins is still limited and new “tetraspanin signalosomes” are waiting to be discovered. Analysis of tetraspanin protein expression in MCs on subcellular level, involvement of further tetraspanins in the regulation of key MC signaling complexes, potential contribution of tetraspanins to MC innate response and tetraspanin-dependent mechanisms involved in the regulation of MC exosome production will be exciting topics for future research. The role of miRNAs and transcriptional regulation of tetraspanin expression, involvement of epigenetic regulation, and the potential roles of various post-translational modifications on tetraspanin functions remain to be investigated. The development of new tetraspanin-specific antibodies and in-depth characterization of MC phenotype and function in tetraspanin-deficient mice, coupled with single-cell-based techniques in human MC analysis, will be the prerequisites for understanding how the members of this membrane protein family are regulating MC function. The knowledge of tetraspanin biology applied for this exceptional cell type could give rise to unexpected discoveries and innovative therapeutic approaches for allergies, asthma, and anaphylactic reactions.
